# Inflammatory Mechanisms of HCC Development

**DOI:** 10.3390/cancers12030641

**Published:** 2020-03-10

**Authors:** Maria Grazia Refolo, Caterina Messa, Vito Guerra, Brian Irving Carr, Rosalba D’Alessandro

**Affiliations:** 1 Laboratory of Cellular and Molecular Biology, Department of Clinical Pathology, National Institute of Gastroenterology, “Saverio de Bellis” Research Hospital, 70013 Castellana Grotte, BA, Italy; maria.refolo@irccsdebellis.it (M.G.R.); caterina.messa@irccsdebellis.it (C.M.); rosalba.dalessandro@irccsdebellis.it (R.D.); 2 Clinical Trial Unit, National Institute of Gastroenterology, “Saverio de Bellis” Research Hospital, 70013 Castellana Grotte, BA, Italy; vito.guerra@irccsdebellis.it; 3 Department of Liver Cancer Biology, Liver Transplant Institute, Inonu University, Malatya 44280, Turkey

**Keywords:** cancer etiology, chronic inflammation, immunosurveillance, immunosuppression, HCC

## Abstract

HCC (hepatocellular carcinoma) is the second leading cause of cancer deaths worldwide, with several etiologic causes, mostly inflammation-associated. Different inflammatory responses in the liver can be triggered by different etiological agents. The inflammatory process can be resolved or be persistent, depending on the etiology and multiple other factors. Chronic inflammation, tissue remodeling, genetic alterations, and modifications in cellular signaling are considered to be key processes promoting immunosuppression. The progressive immunosuppression leads to the inactivation of anti-tumor immunity involved in HCC carcinogenesis and progression. Tumor cellular processes including DNA damage, necrosis, and ER (endoplasmic reticulum) stress can affect both immune-surveillance and cancer-promoting inflammation, supporting a mutual interdependence. Here, we review the current understanding of how chronic liver injury and inflammation is triggered and sustained, and how inflammation is linked to HCC. The identification of many hepatic microenvironmental inflammatory processes and their effector molecules, has resulted in extensive translational work and promising clinical trials of new immunomodulatory agents.

## 1. Introduction

Several lines of evidence led to the consideration that a deregulated microenvironment might be a major factor in tumorigenesis. Chronic inflammation is associated with high incidence of several cancers [1,2]. HCC (hepatocellular carcinoma), which arises mostly in inflamed livers, represents one example of this well studied relationship. Inflammation-inducing factors include HBV (hepatitis B virus), HCV (hepatitis C virus), diabetes, obesity, excessive alcohol consumption and metabolic diseases, which contributing to fibrosis and cirrhosis are considered to be factors that predispose to HCC. In addition, specific features of the hepatic microenvironment exert selective pressure that could explain the diversity found in different types of liver cancer [3]. This review aims to consider several crucial aspects of the liver microenvironment as having a role in driving hepatocarcinogenesis and tumor survival, as well as some new therapeutic approaches deriving from this knowledge [4,5], highlighting the existence of different pathways of carcinogenesis triggered by viral infections with respect to those induced by other etiologic agents.

## 2. Microenvironmental Factors in HCC

Chronic inflammation, tissue remodeling, genetic alterations, and alterations in cellular signaling are considered to be key processes involved in HCC carcinogenesis and progression, and they are driven by liver microenvironmental factors, which in turn are modified by their mutual interdependence.

### 2.1. DNA Alterations in HCC

Each HCC tumor is to be considered as comprising a specific set of somatic mutations including genetic, epigenetic, transcriptomic and metabolic alterations that constitute its unique molecular fingerprint [6]. The spontaneous accumulation of genetic alterations in cancer cells derives from several events such as virus infections, carcinogen exposure (aflatoxin B1) and defects in DNA repair mechanisms. The first studies that identified mutations in HCC are those related to HBV infection that have highlighted the key role in the carcinogenesis process of mutations in b-catenin (CTNNB1) and TP53, occurring in 20%–40% and 20%–50% of cases, respectively [7,8,9,10]. A high frequency of specific TP53 mutations was also revealed after prolonged exposure to aflatoxin B1 [11]. Although the insertion of the HBV virus may occur randomly, it is now widely demonstrated that there are preferential insertion sites, such as TERT, MLL4, CCNE1, CCNA2, and RARB [12,13,14,15]. In some cases, the insertion is associated with the over expression of the corresponding gene [12], in others it is associated with chromosomal instability [15]. Furthermore, viral proteins may exert oncogenic activity on the mechanisms of activation of signaling pathways involved in cell growth and motility [16]. Therefore, HBV infection can determine the onset of HCC even in the absence of inflammation or cirrhosis. On the contrary, in all cases in which the carcinogenesis process takes place in a cirrhotic liver, it is difficult to establish a causal link between a given inflammatory context and the onset of specific mutations in the liver cells. This scenario is typical of HCC related to HCV, alcohol consumption, food toxins or metabolic alterations. Chronic inflammation and oxidative stress likely contribute to further accumulation of genetic alterations in hepatocytes, especially if already in a cirrhotic liver [17]. However, numerous studies have tried to identify possible driver genes even in these contexts. Considering HCV-related HCC, some studies suggested that HCV proteins may exert direct oncogenic activity by interfering with signaling cascades such as the Wnt/B-catenine, TGFB, NFKB or P53 pathway [18,19]. Concerning alcohol consumption acetaldehyde and reactive oxygen species metabolites may induce mutations by binding to DNA or inducing lipid peroxidation and DNA adducts respectively [20,21,22]. Moreover, chronic oxidative stress due to alcohol intake and cytokine release leads to chronic inflammation, cirrhosis, and progression in HCC. Interestingly, polymorphism of superoxide dismutase and MnSoD has been associated with HCC occurrence in alcohol-related cirrhosis, but not in HCV-related cirrhosis [23]. Our knowledge of the cancer genome is significantly improved thanks to genome wide sequencing using NGS (next-generation) technologies [24]. NGS has provided a comprehensive landscape of frequency of specific molecular alterations useful to better understand their molecular mechanisms and to identify the key driver genes involved in HCC pathogenesis and progression [25]. This knowledge will open up new and more effective therapeutic approaches NGS studies revealed that ARID1A and ARID2 are mutated in 15% and 10% of HCC patients, respectively [26]. ARID1A and ARID2 coding for proteins acting as tumor suppressor genes and involved in the SWI/SNF complex which plays a key role in chromatin remodeling and transcription control. In some cases, the ARID1A mutation was associated with high alcohol intake [26] as well as 6p21.1 amplification of FGF19 was described in NASH-derived HCC [27]. Other mutations, identified in KEAP1 and NFE2L2, are involved in constitutive activation of the stress oxidative signaling. Moreover, other less frequent mutations are revealed in some of the gene of the MAPK pathway such as PIK3CA, RAS and RPS6KA3 [28].

Although the improvement in the molecular analyzes of the whole genome, the identification of the possible driver genes is still incomplete. Further efforts will be needed to shed light on NAFLD (non-alcoholic fatty liver disease)/NASH (non-alcoholic steatohepatitis)-related carcinogenesis mechanisms (see also Section 2.6). The analyses conducted at the system biology level are proving to be of great interest. Recently, Chen and colleagues [29] carried out a bioinformatic analysis of 1074 published articles that led to the identification of 560 human genes associated with HCC. These genes are involved in gene expression, protein catabolism, DNA methylation or transcription. Through biological function enrichment analysis, they were also able to identify specific pathways closely related to hepatocarcinogenesis and involved in cellular processes such as apoptosis, necro-apotosis and cell cycle. Based on their crosstalk, these pathways could be divided into three major modules. The first module includes pathways involved in signal transmission, immunological suppression, cellular metabolism and regulation of various hormonal factors; the second module directly associates HBV and HCV viral infection with the onset of HCC; the third module includes genes with altered expression, that act synergistically in the modulation of the same biological function in various cancers. This comprehensive and systematic analysis allowed the identification of 14 genes as potential biomarkers in the diagnosis and treatment of HCC. Of great interest are the CDK2 and CDK4 that cooperate, in association with cyclin E and D, in promoting the transition from phase G1 to phase S of the cell cycle. Therefore, an alteration of these genes directly affects the regulation of the cell cycle during hepatocarcinogenesis. However, TP53 modulating the CDK2-4-inhibitor CDKN1A acts as suppressor of tumorigenesis. TP53 also has an important role in inhibiting the expression of VEGFA and therefore the process of neo-angiogenesis and tumor growth. These genes have a robust “classification effectiveness”, able to distinguish normal samples from tumors. The goal of this study was the identification of potential targets for the diagnosis and treatment of HCC. It is a widespread opinion that somatic insertion and/or deletion mutations involving driver genes can lead to the formation of neo-antigens. Although the presence of neo-antigens stimulates the immune system their prolonged exposure generally causes the establishment of an immune tolerant microenvironment supported by the continuous interaction between PD-1 receptor and its ligand PD-L1 leading to T cells exhaustion [30,31].

Although the lack of drugs targeting the most prevalent mutations represent a crucial limiting step [32], data derived from mutation analysis could also help to predict response to new treatments including immunotherapies, particularly immune checkpoint blockade [33], as well as molecularly-targeted inhibitors of known genetic changes occurring in HCC, such as in the FGFR (Fibroblast Growth Factor Receptor) gene amplification (Blueprint’s BLU-554). Interestingly, response to anti-PD1-L1 (Programmed Death-Ligand1)-based therapies was significantly better in patients with colorectal cancer and mismatch-repair status, which was associated with a high TMB (tumor mutational burden). A high TMB is associated with an increase in neo-antigens and a consequent stimulation of the immune system [34,35]. These conditions seem to correlate with response to immune checkpoint blockade as well demonstrated in different solid tumors [36,37].

### 2.2. Chronic Inflammation

**Figure 1:**
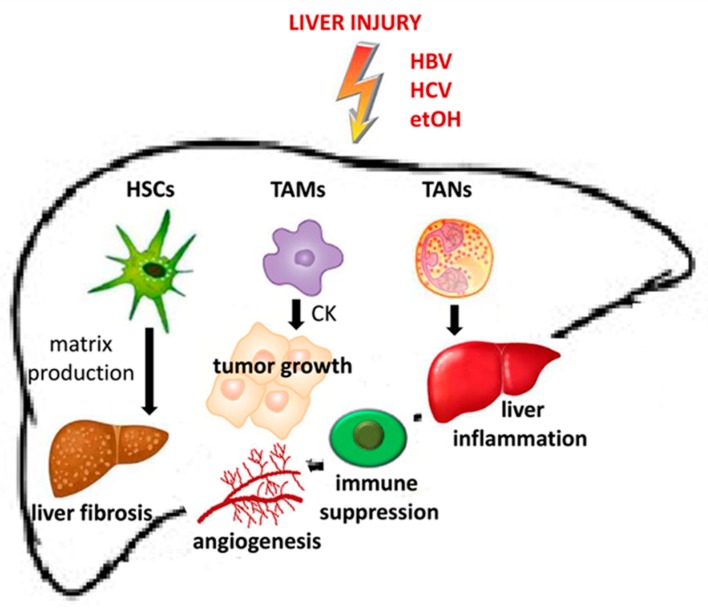
Role of stromal cells in the realization of premalignant microenvironment and HCC onset. HSCs (Hepatic Stellate Cells), TAMs (Tumor Associated Macrophages) and TANs (Tumor Associated Neutrophils) contribute respectively to induce liver fibrosis by matrix production, to promote tumor growth prompting the angiogenesis process and immune suppression leading to liver inflammation.

Chronic inflammation, characterized by infiltration of macrophage and immature myeloid cells and dysregulated production of cytokines, may be considered to be one of the main triggers of HCC carcinogenesis and tumor progression. In the premalignant stage, chronic activation of inflammatory signaling pathways results in the generation of ROS (reactive oxygen species) and NOS (nitrogen oxygen species). Inflammatory cells (including stromal cells) within the premalignant environment produce a vast array of cytokines, growth factors, chemokines, prostaglandins, and proangiogenic factors (Figure 1).

**Figure 2:**
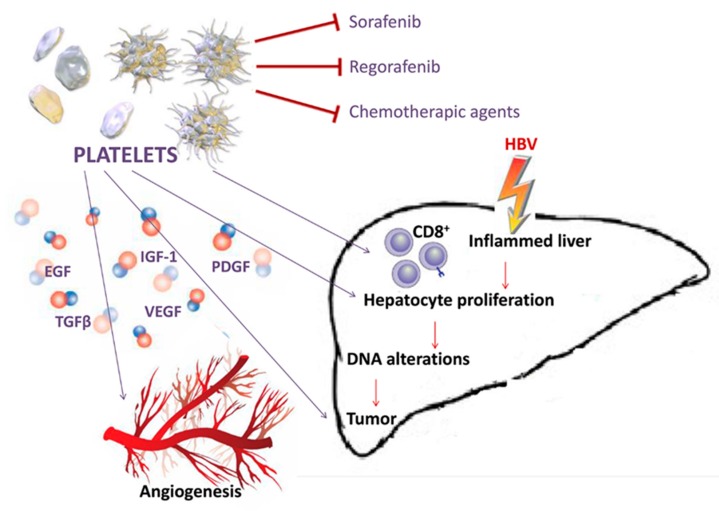
Platelets amplify liver damage. Growth factors released by the activated platelets can reinforce the changes induced by HBV at the level of the hepatic parenchyma as well as the formation of new vessels. Platelets create a microenvironment that opposes the action of drugs such as Sorafenib, Regorafenib and chemotherapic agents.

Altogether these molecules play a key role in creating a microenvironment that supports the transformation of hepatocytes, but also induces their survival through activation of anti-apoptotic pathways and inhibition of immune surveillance. During HBV infection, the accumulation of virus-specific CD8+ T cells and nonspecific inflammatory cells into the liver parenchyma are mediated by platelets which contain several inflammatory molecules and immune mediators, stored in intracellular granules [38]. Platelet activation recruits inflammatory cells to the site of inflammation and induces the expression of several GFs (growth factors) that mediate cellular proliferation and neo-angiogenesis (Figure 2). High levels of platelets and associated GFs detected in thrombocytosis significantly correlate with large size HCCs [39,40]. Both thrombocytosis and large tumor size are reported with a high incidence in association with portal vein thrombosis, metastasis and poor prognosis [41,42]. Platelets and PDGF (platelets derived growth factor) have been described to have a stimulatory effect on growth of normal and tumoral hepatocytes [43,44]. A mechanism by which platelets contribute to liver injury is their integrin-dependent adhesion to LSECs (liver sinusoidal endothelial cells), which are thus activated through NF-kB signaling. Activated LSECs secrete specific chemokines that regulate the recruitment and adhesion of leukocytes to the endothelium [45]. Furthermore, in the context of hepatitis B infection, infected hepatocytes are killed by CD8+ T cells adherent to the sinusoids in a platelet-dependent process [46].

Moreover, growing data show that the modulatory actions of platelet factors such as EGF (epidermal growth factor), IGF-I (insulin-growth factor-I), TGF-β (transforming growth factor beta), PDGF and VEGF (vascular endothelial growth factor) alter the cancer chemotherapy sensitivity or resistance [47]. Our recent results supported a primary role of EGF and IGF-I in interfering with the inhibitory action of Sorafenib and Regorafenib in HCC cell lines [48,49,50] (Figure 2).

As a consequence of increased proliferation of hepatocytes, shortening of telomeres and the consequent chromosomal instability was observed in 90% of HCC carcinogenesis and progression [51]. Mechanisms of telomerase reactivation in HCC are associated to TERT promoter mutations, TERT (telomerase reverse transcriptase) amplification, chromosome translocations and HBV or adeno-associated virus type 2 insertion into the TERT promoter [51,52,53]. An important risk factor for the occurrence of HCC is given by the replacement of hepatocytes with liver progenitor cells that occurs following a chronic infection. The presence of pro-inflammatory cytokine such as IL-6 (Interleukin-6) enhances the transformation of the liver progenitor cells into a more cancerous phenotype [54] (Table 1).

The chronic inflammation plays also a key role in the mechanisms leading to NASH-derived HCC. Obesity or other genetic factors contribute to the development of insulin resistance and steatosis. The metabolic alterations occurring in injured hepatocytes trigger the inflammatory response mainly through the pathways activated by the Toll-like receptors. Although “senescence surveillance” pathways [55] (Table 1), involving p53 and N-Ras, directed to remove cancer cells are activated, other mechanisms intrinsic to the cancer cells together with microenvironmental factors may be related to immune system dysfunction. Hepatocytes actively recruit Kupffer cells as well as other components of the innate immune response through the activation of the inflammasome and the coordinated release of pro-inflammatory and pro-fibrogenic cytokines and ligands.

These considerations support the interest in the use of nonsteroidal anti-inflammatory drugs (NSAID) as potential agents to reduce chronic risk of neoplastic progression due to their anti-inflammatory properties. These drugs, especially aspirin, are able to reduce cellular growth acting on COX (cyclooxygenase) enzymatic pathways, which mediate inflammation [56]. Each category of NSAID may represent different levels of selectivity for COX isoforms and different mechanisms of action [56,57] (Table 1). Although there is growing literature concerning the role of NSAIDs in cancer prevention, the results can be contradictory. This is probably due to the poor knowledge of the molecular mechanisms underlying the beneficial or side effects of these drugs in each type of cancer, including HCC [58].

### 2.3. Inflammation and Tissue Remodeling

Chronic inflammation also leads to tissue remodeling through the crosstalk among several cell types in the liver microenvironment. Stromal cells, including fibroblasts and HSCs, can enhance ECM (extra cellular matrix) synthesis [66]. The immune cells can contribute to ECM remodeling through the activation of stromal cells or the synthesis of MMPs (matrix metallo peptidases) that specifically cleave ECM modifying the structure and function of the HCC microenvironment. With positive feed-back loop, the activated HSCs, but also Kupffer cells, macrophages and platelets are responsible for the secretion of TGF-β that in turn activates more HSCs. As a result, the production and the release of ECM molecules, tissue MMPs, such as MMP2, 9, and 13, and MMPs inhibitors, such as TIMP1, are increased [67,68,69]. Tumor cells can induce remodeling by stromal cells through altered signaling pathways. ECM degradation allows tumor growth as well as facilitates the release of GFs and may also generate bioactive cleavage products or cell surface receptors, all of which represent pro-proliferative signals for tumor cells. Moreover, ECM degradation disables the growth-suppressing adhesion complexes favoring tumor cell evasion of growth suppression [70]. The remodeling that occurs at the ECM level is closely related to alterations in blood flow and the consequent hypoxia, and all of these processes ultimately favor tumor progression. In general, the cellular response to hypoxia consists of an increased expression of pro-angiogenic factors in tumor and non-tumor tissues. In particular there is an upregulation of VEGF which exerts its action at the level of hematopoietic stem cells and liver endothelial cells, which respond by forming new vessels that compensate for the lack of oxygen and nutrients necessary for tumor growth [71,72]. The resulted neo-angiogenesis is insufficient to compensate for the tumor hypoxia, consequently the remaining hypoxic areas in turn allow the formation of new vessels and the amplification of the angiogenic process [73,74]. These areas of hypoxia can also lead to molecular changes in signaling in non-tumor cells that modulate the HCC progression [75]. Moreover, it is known that regions of hypoxia are sites of accumulation for macrophages that in this condition assume a phenotype associated with increased expression of tolerogenic molecules, such as PD-L1 and IL-10, as well as pro-angiogenic factors, such as angiopoietin 1, VEGF, and MMP-9 [76]. One of the key signals that are activated by hypoxia is represented by HIFs (hypoxia-induced factors) which in turn activate the transcription of genes involved in EMT (epithelial-mesenchymal transition), invasion of the extracellular matrix and in other processes leading to tumor spread. Other findings revealed the existence of HIF-independent regulation of tumor angiogenesis and chemo-resistance under hypoxic conditions [77]. Furthermore, several studies explored the role of signaling pathways such as YAP (yes associated-protein), MMPs, HMGB1 (high mobility group box 1) and glucose metabolism enzymes in mediating the effects triggered by hypoxia in HCC [59] (Table 1). Guise C.P. and colleagues also described the hypoxia-activated bio-reductive pro-drugs, which would be activated by enzymatic reduction under hypoxic conditions [78]. An attractive possibility consists in targeting key molecules specifically induced in hypoxic cells, such as the HIF inhibitors (HIF1α mRNA antagonist RO7070179) or molecules that specifically target the hypoxic fractions of tumors such as HAPs (hypoxia-activated prodrugs). These drugs are being administrated in combination with Sorafenib in ongoing phase I/II trials (clinicaltrials.gov Identifier: NCT02564614, NCT01497444 and NCT00862082). In addition, strategies such as oxygen supplement [79] and vessel normalization [80] could represent an effective option in the treatment of hypoxic cancers.

### 2.4. Inflammation and the Immune Response in HCC

From the interaction between the immune system, tumor cells and microenvironment derive a specific relationship between pro-tumor and anti-tumor factors, which changes over time and can either result in tumor elimination or elicit tumor progression. All tumors could indeed be considered potentially immunogenic and the host immune system is able to generate T cell responses that recognize and eradicate cancer cells. Several immune check point factors, including CTLA-4 (cytotoxic t-lymphocyte antigen-4) and programmed cell PD-1 (programmed death-1), downregulate the amplitude of immune response participating in peripheral self-tolerance. Cancer can use these signaling pathways leading to overexpression of check point molecules. One of the major effects of this upregulation is the inhibition of T cell activity (CD8+ effectors and CD4+ helpers which integrate adaptive and innate effector mechanisms) and the ensuing suppression of tumor immunity as a major mechanism of immune resistance. Moreover, the continuous interaction among tumor cells, immune cells and TME (tumor microenvironment), significantly contributes to these resistance mechanisms [81]. It is widely accepted that the TME of HCC is strongly immunosuppressive, thus resulting in immune tolerance and evasion by different mechanisms. A comprehensive integrated analysis of HCC conducted by the Cancer Genome Atlas Research Network revealed that approximately 25% of HCC cases present high inflammatory scores, with high or moderate levels of lymphocyte infiltration [82]. Although the high density of TILs (Tumor-Infiltrating Lymphocytes) is normally associated with a response of the host immune system against cancer and better outcome [83], this cellular response can be dysfunctional with a higher proportion of CD4+ (helper or T regulatory cells) to CD8+ cells. Major suggestions also indicate that HCC tumor cells may directly modify the liver microenvironment by recruiting Treg (regulatory T-cells) [84,85]. This scenario induces immune tolerance and could be associated to worse prognosis [86]. Moreover, TILs can be also found in the premalignant status of cirrhosis, but they were shown to be insufficient to suppress tumor progression [87]. The cytotoxic activity of CD8+ T cells are impaired though several mechanisms involving tumor cells, inflammatory cells and stromal cells. TAFs (Tumor-Associated Fibroblast), including HSCs, can promote the differentiation of peripheral blood monocyte into MDSC (myeloid derived suppressor cells) through IL-6/STAT3 signaling [88]. Both TGF-β and cytokines (such as IL-10), secreted by HSCs, Kupffer cells, TAMs (tumor associated macrophages), and platelets, determine an expansion of MDSCs which in turn play a key role in enabling the effector T cells, enhancing the immune check-point signaling and decreasing NK (natural killer) cell cytotoxicity and cytokine production [89,90]. The presence of these immunosuppressive cells correlates with reduced TILs, elevated tumorigenicity, aggressive phenotype in HCC patients [91]. Moreover, MDSCs can contribute to the creation of an immunosuppressive TME, by inducing activation of Treg cells and inhibiting the secretion of IFN-ɣ (Interferon gamma) by NK cells [92]. MDSCs also compete with T cells for energy resources (e.g., arginine and cysteine) [93]. The binding of the ligand galectin-9 on MDSCs with the receptor TIM-3 (T cell immunoglobulin and mucin domain-containing protein 3) on T cells determine their apoptosis [94]. Furthermore, in advanced HCC it has been hypothesized that MDSCs may interact with Kupffer cells to enhance PD-L1 expression, thus inhibiting cytotoxicity and cytokine release by NK cells [95]. Similarly to TILs, also for other immune cells, such as TAMs and tumor-associated neutrophils (TANs), recognition of pro-tumor and anti-tumor subtypes has been shown in several studies [96,97,98]. HCC cells play a key role in inducing the differentiation of circulating monocytes in M2 pro-tumor macrophages through the secretion of specific cytokines and growth factors, such as IL-4, IL-13, CSF-1 (Colony Stimulating Factor-1), CTGF (Connective Tissue Growth Factor) [60,99] (Table 1). Moreover, Osteopontin, secreted by HCC cells, induces both PD-L1 expression and attraction of M2 in the tumor site [100]. TAMs together with MDSCs, through the down regulation of immune-permissive IL-6 and IL-12 and over-expression of immune-suppressive IL-10, reduce both innate and adaptive immunity by impairing CD8+ T cells and NK cells respectively [101]. Similarly to TAMs, pro-tumoral N2 TANs are activated by IFNs and TGF-β, and induce apoptosis of CD8+ T cells via TNF alpha-mediated nitric oxide production [102,103]. Recently, TANs regulatory T cells are been associated to HCC Sorafenib-resistance and progression [104]. Recently, Liao et al. also suggested a cooperation of PD-1 and other immune checkpoint pathways that allows the escape of cancer cells from the control operated by the immune system [31]. This tolerant immune microenvironment is also attributed to an imbalance between pro-inflammatory and anti-inflammatory cytokines in HCC, closely correlating with increased PD-L expression. Thus, the development of ICI (immune checkpoint inhibitors) able to induce the immune system to attack cancer cells has a key role in the development of new therapeutic strategies against cancer. The expression of PD-L1 at the level of tumor cells is considered the main factor for the identification of the patients who may benefit from PD-1 axis targeted therapy. However, a significant percentage of patients with this positivity do not respond to these therapies, suggesting the existence of other interactions that are independent of PD-L1. Interesting results revealed that the immune-responsive signature is related to early stage HCC and could be associated with favorable prognosis and response to ICIs therapies [105]. This idea was supported by other studies that showed an initial prevalence to immune response against tumor antigens followed by dysfunctional effector cells in later stages of HCC [106]. Thanks to new technologies, efforts are being made to correlate the probability of transition from an inflammatory state to a tumor with a given immune arrangement in the liver. Integrated data derived from transcriptomic signature and immunohistochemistry open new perspectives for stratification of patients who might benefit for long-term from immune checkpoint therapy or other therapeutic approaches overcoming the immunosuppressive and dysfunctional HCC immune system [107].

### 2.5. Other Microenvironmental Factors Involved in Hepatocarcinogenesis

New areas of research indicate other aspects of the microenvironment, such as diet, gut microflora and micro-vesicles derived from cell subpopulations within the liver, in HCC carcinogenesis and progression [108,109,110,111]. Although it is currently difficult to define the exact causal mechanisms, these aspects warrant further investigation in greater detail in coming years. High fat diet-related microbial products, deriving from the gut, actively modulate a senescence-associated secretory phenotype on HSCs, with the ensuing production of PGE2 (prostaglandin E2) and inhibition of NK and cytotoxic T cells. Cytotoxicity may also be reduced by the effects of Gram-positive bacteria on the bile acid metabolism thus decreasing CXCL16 expression by LSECs [61,62,63,112].

### 2.6. HCC Etiology and Chronic Inflammation

**Figure 3:**
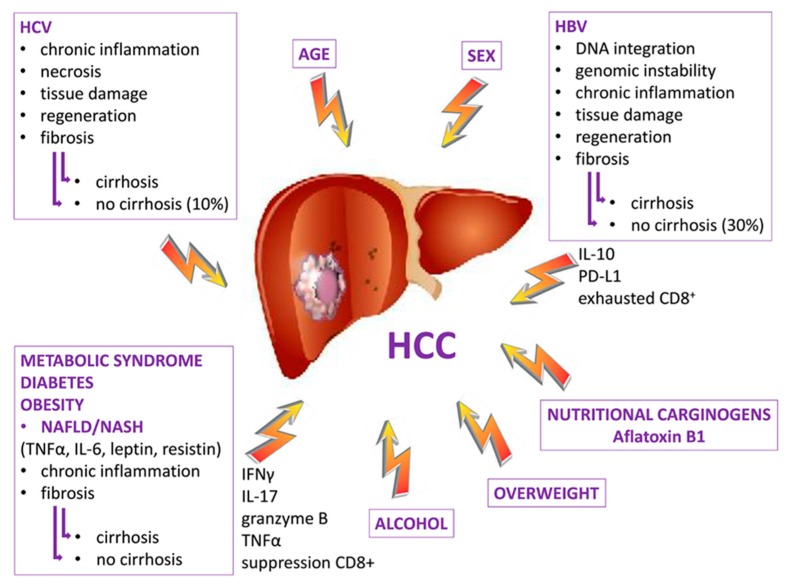
Cooperating risk factors for HCC onset. Factors with a viral and non-viral etiology may be responsible for the onset of the HCC by releasing specific mediators that lead to the formation of an inflammatory microenvironment that predisposes to the formation of fibrosis which can develop into cirrhosis which in some cases is not an essential condition for the development of HCC.

The 80%–90% HCC of cases occur in the setting of chronic inflammation due to various etiological agents, such as toxins, viruses, excessive alcohol consumption and NAFLD/NASH. In these contests the cancer can represent the evolution of a fibrotic process in the presence or absence of cirrhosis (Figure 3).

However, it will be considered also HCC "de novo" occurring in a little percentage of patients (10%–20%) without concomitant fibrosis/cirrhosis and therefore without chronic inflammation [113]. Considering most inflamed-HCC, the progression of the fibrotic process is associated with an altered hepatic structure and an even greater alteration of immunological proteins important for the control of inflammation of the healthy liver, such as the C3 and C4 proteins, that appear strongly down regulated in the cirrhotic liver [114,115]. As a general rule, increasing inflammation is associated with a microenvironment with enhanced immunosuppressive signature. The chemokine networks CXCR3/CXCL10 and CCR6/CCL20 [116,117] together with TGFβ [64] (Table 1) play a key role in orchestrating the recruitment of Treg and macrophages polarized in their immunosuppressive phenotype M2 [65] (Table 1). Kupffer cells produce also enhanced levels of PD-L1 expression [118]. The absence of cirrhosis is found primary in 30% of HCC derived from HBV infection and in variable percentage of HCC derived from NAFD/NASH, but also in about 10% of HCV infection. In chronic HBV infection, high viral load, mutation BCP T1762/A1764 or overweight are considered risk factors for the occurrence of HCC even in the absence of cirrhosis [119,120]. In chronic HCV infection, cell death is largely due to inflammation and necrosis resulting in tissue damage, regeneration and fibrosis. The absence of cirrhosis in the HCV-related tumors is found after virus eradication treatment and in the presence of other risk factors that are in any case responsible for the inflammatory state [121,122]. Growing evidences revealed higher levels of IL6, TNFα, IL1β, and IL10 in HCC patients with cirrhosis compared with those infected with HBV or HCV in the absence of cirrhosis [123,124,125].

Although innate and adaptive immune responses restrict HBV replication, chronic viral infection promote inflammation together with a program that support HCC progression through immunosuppressive TME, consisting in IL-10, PD-L1 expression and exhausted CD8+ T cell [126]. Hwang IC and colleagues proposed the existence of different pathways of carcinogenesis triggered by viral infections respect to those induced by other agents [127]. They conducted a population-based cohort study on 2,336 HCC Korean patients in which the risk of HCC occurrence was related to the aspirin assumption. Aspirin inhibits COX, thus affecting platelet action that, as previously mentioned, plays a pivotal role in inducing the immune-mediated liver inflammation. The results revealed a protective role exerted by aspirin in patients with HBV infection without cirrhosis, in which platelets may promote the inflammation immune-mediated triggered by chronic viral infection [38]. In this contest, the risk of HCC was significantly lowered by aspirin. If further studies confirm these results, aspirin could be used to lower the risk of HCC in a selected patient population.

Moreover, a well-known link was established between inflammation and epigenetic mechanisms and the comparison of methylation profiles between patients with HBV infection, HCV infection and alcohol consumption has revealed a specific set of hypermethylated CpG islands for each group [128]. Recent integrated studies highlighted other interesting correlation between immune landscape, including PD-L1/PD-1 expression, and HCC etiopathology. In this regard, the results based on the analysis of 956 patients showed a closer relationship between inflammatory phenotype, PD-L1 overexpression and HCV infection compared to that observed in case of HCC deriving from HBV or excessive alcohol consumption [129]. Further efforts will be needed to extend these analyzes to include HCC cases resulting from NAFLD/NASH. With the decrease in HCC cases due to virus eradication treatments, excessive alcohol consumption and NAFLD/NASH represent the main persistent risk factors of HCC in the Western world. The presence of NAFLD, which is associated with type 2 diabetes and obesity, causes the release of numerous inflammatory factors, such as TNF-alpha, IL-6, leptin, and resistin, that are at the base of the steatosis process and that promote tumorigenesis [130]. In a recent study, aimed to elucidate the molecular mechanisms involved in the development of NASH, a transcriptional and immune profiling of NASH patients before and after LSI (lifestyle intervention) was performed [131]. The results revealed a hepatic gene signature associated with NASH regression due to LSI independently of body weight loss. The genes specifically associated with NASH included loci linked to inflammatory responses, antigen presentation and cytotoxic cells. Furthermore, analysis in an independent cohort showed a close link between NASH and alterations in blood immune cell types, including cDC (dendritic cells) type 1 and 2, and cytotoxic CD8+ T cells. The hepatic expression levels of some of these immune-related genes, such as CXCL9 and CXCL10, and lysozyme, were significantly higher in NASH respect to NAFLD patients. Blood cytotoxic CD8+ T cells expressing perforin showed associations with hepatic genes related to cytotoxic and IFNγ responses (GZMA, CD226, IRF1), T helper differentiation (ITK), and TNFα signaling (TNFAIP2). Interestingly, these results found that disease activity, lobular inflammation and ballooning in NASH is related to the accumulation of CD8+ T lymphocytes in the liver. Hepatic CD8+ T cells are also linked to an enhanced expression of genes from the signature of active NASH. In NAFLD/NASH-related HCC tumors were found increased levels of IgA-producing cells that induce tumorigenesis though PD-L1 and IL-10 expression and the suppression of CD8+ T cells [132]. Generally, in non-viral HCC patients were found higher levels of IFNɣ, IL-17, Granzyme B, and TNFα with respect to those revealed in HBV-related patients, thus explaining the more immunosuppressive environment created by viral infection [126].

## 3. Preclinical Studies in HCC Immunotherapy

Due to the lower response rate to ICB (immune-checkpoint blockade)-based immunotherapies in preclinical studies, ICB agents were also tested in combination with other systemic treatments. Chen Y and collaborators showed that the prevention of an immunosuppressive microenvironment, through CXCR4 inhibition, facilitated the response to anti PD-1 therapy in HCC mouse models previously treated with Sorafenib [133]. Most recently, using both subcutaneous and hepatic orthotopic models, Brown et al. provided evidence that inhibiting IDO in combination with ICIs could add therapeutic benefit in tumors overexpressing IDO [134]. Considering that angiogenesis inhibitors and ICIs are used for anticancer therapies against advanced HCC, Kimura T and colleagues also reported that VEGFR inhibitor Levantinib enhanced the antitumor activity of PD-1 blockade, decreasing the proportion of the monocyte and macrophage population and increasing CD8+ T cell populations in the Hepa1-6 mouse HCC syngeneic model [135].

The activity of immunotherapeutic agents may also be improved by epigenetic drugs that have immune-mediated antitumor effects. This hypothesis was studied by Llopiz D et al., who investigated the therapeutic efficacy of anti-CTLA-4 and anti-PD-1 antibodies in combination with the HDAC (histone deacetylase inhibitor) inhibitor Belinostat in a subcutaneous Hepa129 HCC model. This drug combination resulted in an improvement of the antitumor activity of anti-CTLA-4 but not of anti-PD-1 therapy [136]. Moreover, preclinical data suggested that polyIC (polyinosinic-polycytidylic acid) represents an efficient prevention strategy against liver tumorigenesis and a powerful combination immunotherapy for HCC activating NK cells, macrophages and some T-cell subpopulation [137]. The expansion of TAMs in the TME allows the immune escape of cancer cells. Therefore, strategies aimed to prevent TAM trafficking enhanced the efficacy of ICI in HCC mouse models [101]. Other strategies targeting immunosuppressive cells have been tested in the preclinical setting. The FDA-approved MDSCs inhibitor Tadalafil may enhance the Cytokine-induced killer (CIK) cell-based immunotherapy in HCC patients [138].

Murine HCC models have also been used to investigate the ability of vaccines to trigger a specific response by T cells and to assess the efficacy of CAR-T (chimeric antigen receptor T) cells. Recently, Liu Z et al. investigated the effects of the fusion of the potent immune-adjuvant HMGN1 (high-mobility group nucleosome binding protein 1) to AFP in a AFP-expressing lentiviral vector (lenti-HA). This vehicle was extensively employed for genetic immunization with high transduction efficacy and a good safety profile. The antitumor immunity of Lenti-HA was assessed in ectopic, orthotopic, and autochthonous HCC models, demonstrating that HMGN1 increased the antitumor immunity of AFP-expressing lentiviral vaccines in HCC mice and human cells, and thus providing a new therapeutic strategy for HCC [139]. Moreover, several findings demonstrated that CAR T-cell immunotherapy targeting intracellular/secreted tumor antigens can elicit a potent antitumor response. The effects of CAR-T cells targeting AFP (alpha-fetoprotein) or GPC3 (glypican-3) were also evaluated with promising results in HCC patients [140,141].

## 4. Clinical Studies in HCC Immunotherapy

The inhibitors of immune check points are currently investigated in clinical trials alone or in association with local regional interventions or standard therapies involving chemotherapy or molecularly targeted drugs. Tremelimumab, a monoclonal antibody against CTLA-4, was the first ICB drug tested in HCC patients. NCT01008358 clinical trial showed that this drug had a satisfactory safety profile and produced 17.6% partial response in evaluable patients.

Considering the PD-1 inhibitors, Nivolumab was administrated to patients with advanced HCC and poor survival prospects in a phase I/II trial (NCT01658878). The ORR (objective response rate) of 15–24% and promising survival durations led to the approval by the U.S. FDA. Pembrolizumab, also received FDA approval following the results obtained from NCT02702414 phase II study that reported one complete response and 16 partial responses among 104 patients and found drug effectiveness and tolerability. Pembrolizumab has also been evaluated in two other randomised, phase III trials as a second-line HCC treatment (NCT02702401 and NCT03062358). However, data from NCT02702401 study revealed that co-primary endpoints of OS (overall survival) and PFS (progression-free survival) were not achieved in advanced HCC (Table 2).

The evaluation of PD-L1 expression levels is useful to predict the response to a therapeutic regimen based on ICB drugs. In phase III studies, Durvalumab (anti-PD-L1) monotherapy produced a major promising response rate in HCV-positive HCC patients expressing higher levels of PD-L1 [30,142] (Table 3).

In addition, several ongoing studies are based on therapeutic regimens in which ICB drugs are administered in association with tyrosine kinase inhibitors, anti-VEGF antibodies and ablative interventions. NCT01658878 study (Table 2) showed that blocking simultaneously both PD-1/PD-L1 and CTLA-4 with Nivolumab and Ipilimumab respectively, an average ORR of 31% and an acceptable safety profile were produced in Sorafenib-treated advanced HCC patients. Double blockade with Durvalumab and Tremelimumab was tested in NCT02519348 phase I/II trial with positive results, achieving an ORR of 25% with a manageable toxicity profile. A global phase III trial (NCT03298451) was activated to compare the efficacy of different regimens including Durvalumab as monotherapy, Durvalumab/Tremelimumab combination therapy and Sorafenib monotherapy as first-line treatment in HCC patients (Table 3).

Other combined therapies are being tested in current clinical trials and appear to be promising. Several studies evaluated the simultaneous administration of ICB therapy with targeted drugs such as Atezolizumab (anti-PD-L1) plus Bevacizumab (anti-VEGF) (NCT02715531) (Table 3), Pembrolizumab plus Lenvatinib (a multi-VEGFR inhibitor) (NCT03006926 and NCT03713593) (Table 2), and SHR-1210 (anti-PD-1) plus Apatinib (VEGFR2 inhibitor) (NCT04191889) (Table 4).

Moreover, the global phase III trial NCT03434379 (Table 3) has been recently launched to compare the survival outcome of Atezolizumab and Bevacizumab combination with that of Sorafenib monotherapy as first-line setting for advanced or metastatic HCC.

Enhanced and synergistic effects can also be hypothesized following treatments that provide immune-checkpoint inhibitors combined with locoregional intervention. A phase II trial of Nivolumab in association with mRFA (monopolar radiofrequency ablation) therapy (NCT03630640) proposed to test the safety and efficacy of treatment with Nivolumab in neoadjuvant and adjuvant setting in patients with advanced HCC treated by electroporation in curative intent. Similarly, NCT03337841 study is evaluating the efficacy of the immune checkpoint inhibitor Pembrolizumab in preventing the recurrence of HCC when administered before and after curative surgery or ablation (Table 2).

Furthermore, cell-based immunotherapies exploiting the ability of certain cellular components to reactivate the immune response and the major trials are listed in Table 5.

Results from a phase I study evaluating GPC3 CAR-T cells in refractory or relapsed GPC3+ patients showed a slowdown in tumor progression (NCT02395250). Therefore, interesting clinical trials (NCT02905188 and NCT03198546) are now evaluating the safety and efficacy of T cells genetically engineered with a GPC3-CAR in patients with HCC. Interestingly, the possibility of infusing patients with anti-tumor immune cells such as NK and cytokine-induced killer cells, represents a new therapeutic approach studied in numerous clinical trials (NCT03093688, NCT03319459, NCT02886897).

Due to the ability to specifically infect and kill cancer cells, several onco-lytic viruses have been tested in clinical trials of patients with HCC (Table 6).

The recent NCT02562755 randomized phase III study determines whether treatment with vaccinia virus-based immunotherapy (Pexa-Vec) followed by Sorafenib increases survival compared to treatment with Sorafenib alone in patients with advanced HCC who have not received prior systemic therapy.

## 5. Conclusions

We reviewed multiple aspects of the role of the liver microenvironment and the genetic alterations in hepatocytes cells in relation to HCC development. A multitude of cross talking factors in the liver microenvironment modulate the progression of HCC and their full characterization is crucial to identify new therapies effective in the treatment of the tumor itself and the liver microenvironment, which reciprocally influence each other, to avoid the recurrence and resistance phenomena associated with current HCC therapies. As a result, many microenvironment inflammatory factors have been identified and are potential therapeutic targets. Many immunomodulatory drugs are currently in HCC clinical trials, either alone or in combination with other HCC therapies.

We conclude that the inflammatory response is an active process that not only responds to carcinogenic events, but actively participates in them and is a promising target in its own right for new therapies.

## Figures and Tables

**Table 1:** Experimental evidence linking inflammation to HCC development.

Animal Model	Results	Reference
MUP-uPA transgenic mice	HCC progenitor cells showed autocrine IL-6 signaling that stimulates in vivo growth and malignant progression	[54]
C.B-17 wild-type mice with an intact immunity, C.B-17 SCID mice with an impaired adaptive immune response, C.B-17 SCID/beige mice with defects in NK-cell and macrophage function	Impaired immune-mediated clearance of pre-malignant senescent hepatocytes secreting chemo- and cytokines resulted in the development of murine HCCs	[55]
Rat model of choline-deficient, L-amino acid-defined diet (CDAA)-Male Sprague-Dawley rats Nude mice	Aspirin or nimesulide administration decreased the number of preneoplastic and neoplastic nodules Celecoxib treatment was highly effective in inhibiting the multiplicity and size of liver preneoplastic lesions COX (cyclooxygenase)-2 inhibitors (celecoxib and meloxicam) enhanced tumor cell apoptosis and reduced proliferation	[57]
IL-6–/–/TLR-4–/– C57BL/6J mice	Hepatic stem/progenitor marker CD133 was responsible for driving and maintaining HCC. CD133 expression can be induced by IL-6 and hypoxic conditions in a STAT3-dependent manner	[59]
RAW264.7-shNC/shAIF1 cells and Hepa1–6 cells injected in C57BL/6 mice	Mouse cytokine antibody array analysis showed that macrophages overexpressing AIF1 (Allograft Inflammatory Factor 1) secreted high levels of CXCL16, which is reported to facilitate the migration and invasion of HCC	[60]
C57/BL6 mice	The gut microbiota–driven COX-2 pathway produced PG (Prostaglandin)-E2 which plays a pivotal role in suppressing antitumor immunity and promoting HCC onset	[61]
C57BL/6 mice	Obesity induced alterations of gut microbiota increasing the levels of DCA (deoxycholic acid); the enterohepatic circulation of DCA provoked senescence-associated secretory phenotype in hepatic stellate cells, which in turn secreted inflammatory and tumour-promoting factors in the liver facilitating HCC development	[62]
C57BL/6 mice	Pro-inflammatory conditions enhanced the migration of PGE2 carried by nanoparticles from the intestine to the liver, where they induced the inactivation of natural killer T cells (cancer cells escape from immune control)	[63]
Tak1ΔHep mice	TGF-β promoted HCC development by inducing hepatocyte apoptosis and compensatory proliferation in early phases of tumorigenesis, and inducing expression of anti-apoptotic, pro-oncogenic and angiogenic factors during tumor progression.	[64]
BALB/c mice	Tim-3 (immune regulator, involved in many inflammation-related diseases) expression in tumor-associated macrophages promoted HCC growth	[65]

**Table 2:** Major trials in HCC involving anti-PD-1/PD-L1 agents-based immunotherapies.

Name	Phase	Line of Treatment	Strategy	Primary Endpoint
NCT03841201	2	I	Nivolumab (anti-PD-1/PD-L1) + Lenvatinib (VEGFRs inhibitor)	ORR (Objective response rate) Safety and tolerability
NCT03630640	2	neo + adj	Nivolumab	RFS (Recurrence Free Survival)
NCT02576509	3	I	Nivolumab vs. Sorafenib (Raf inhibotor)	OS (Overall Survival)
NCT03203304	1	II	Nivolumab Ipilimumab (anti CTLA-4)	AEs (Adverse Events)
NCT01658878	1/2	I	Nivolumab Sorafenib Nivolumab + Ipilimumab Nivolumab + Cabozantinib (RTK inhibitor) Nivolumab + Ipilimumab + Cabozantinib	AEsORR
NCT03510871	2	neo	Nivolumab + Ipilimumab	Tumor shrinkage
NCT03841110	1	II	FT500 (NK cell product) +/− Nivolumab, Pembrolizumab (anti-PD-1/PD-L1), Atezolizumab (anti PD-L1), Cyclophosphamide, Fludarabine	DLT (Dose Limiting Toxicities)
NCT03682276	1/2	I	Ipilimumab + Nivolumab	Delay to surgery AEs
NCT03228667	2	II	ALT-803 (IL-15 superagonist) + Pembrolizumab ALT-803 + Nivolumab ALT-803 + Atezolizumab ALT-803 + Avelumab (anti PD-L1)	ORR
NCT04134559	2	II	Pembrolizumab	irBOR (immune-related Best Overall Response)
NCT02595866	1	II	Pembrolizumab	AEs ECIs (Events of Clinical Interest)
NCT03337841	2	neo + adj	Pembrolizumab	One-year RFS
NCT04099277	1	II	LY343515 1+/− Pembrolizumab	DLT
NCT03222076	2	neo	Nivolumab +/− Ipilimumab	AEs
NCT03383458	3	adj	Nivolumab	RFS
NCT03655002	1	II	Nivolumab, Cyclophosphamide, IRX-2 (cytokine-based biologic agent)	Safety
NCT03812562	1	I	Yttrium Y 90 glass microspheres, Nivolumab	RR (Recurrence Rate)
NCT03867084	3	adj	Pembrolizumab	RFS OS
NCT03755739	2/3	I	Pembrolizumab	OS
NCT02702401	3	II	Pembrolizumab	PFS OS
NCT03062358	3	II	Pembrolizumab	OS
NCT02702414	2	II	Pembrolizumab	ORR
NCT03006926	1	II	Pembrolizumab + Levantinib	AEs DLT
NCT03713593	3	I	Levantinib +/− Pembrolizumab	OS
NCT02940496	1/2	II	Pembrolizumab Pembrolizumab + elbasvir/grazoprevir + ribavirin (antiviral drugs)	DLT
NCT03511222	2	I	Vorolanib (antiangiogenic agent) + Pembrolizumab	RP2D (Recommended phase II dose)
NCT03299946	1	neo	Cabozantinib + Nivolumab	AEs proceed to surgery
NCT03412773	3	I	Tislelizumab (anti PD-1/PD-L1)	OS ORR

**Table 3:** Major trials in HCC involving anti-PD-L1 agents-based immunotherapies.

Name	Phase	Line of Treatment	Strategy	Primary Endpoint
NCT03638141	2	II	Durvalumab (anti PD-L1)+ Tremelimumab (anti CTLA-4)	ORR (Objective Response Rate)
NCT03298451	3	I	Durvalumab +/− Tremelimumab	OS (Overall Survival)
NCT03847428	3	I	Durvalumab + Bevacizumab (anti-VEGFA)	RFS (Recurrence-Free Survival)
NCT03434379	3	I	Atezolizumab (anti PD-L1) + Bevacizumab	PFS (Progression-Free Survival) OS
NCT02715531	1	I	Atezolizumab + Bevacizumab	AEs (Adverse Events) OR (Objective Response) PFS
NCT03755791	3	I	Cabozantinib (RTK inhibitor) + Atezolizumab	PFS OS
NCT03937830	2	II	Durvalumab + Bevacizumab + Doxorubicin (TACE)	PFS
NCT02519348	2	II	Durvalumab +/− Tremelimumab Tremelimumab +/− Durvalumab Durvalumab +/− Bevacizumab	AEs DLT (Dose Limiting Toxicity)

**Table 4:** Major trials in HCC involving anti-PD1 agents-based immunotherapies.

Name	Phase	Line of Treatment	Strategy	Primary Endpoint
NCT03864211	1/3	II	Toriplimab (anti-PD-1)	AEs (Adverse Events) ORR (Overall Response Rate)
NCT03914352			SHR-1210 (anti-PD-1)	OS (Overall Survival) DFS (Disease-Free Survival)
NCT03605706	3	I	SHR-1210 + FOLFOX4 FOLFOX4 Sorafenib (Raf inhibitor)	OS
NCT04191889	2		FOLFOX + Apatinib (VEGFR-2 inhibitor) + Camrelizumab (SHR-1210)	ORR
NCT04152356		I	PD-1 + Sorafenib	DFS
NCT04220944	1	I	Sintilimab (anti-PD-1)	PFS (Progression Free Survival)
NCT04174781	2		Sintilimab	PFS
NCT04167293	2/3	I	Sintilimab	24-week PFS
NCT04229355	3	I	Lenvatinib (VEGFRs inhibotor)vs. PD-1 inhibitor	PFS
NCT03949231	3		Toripalimab	OS
NCT03966209	1	II	JS001 (PD-1 inhibitor)	Adverse Events Rate Graft Rejection Rate
NCT03655613	1/2	II	APL-501 (PD-1 inhibitor) + APL-101 (c-Met inhibitor)	DLT (Dose Limiting Toxicity)
NCT04172506	1/2	II	AK105 (anti PD-1)	ORR
NCT03680508	2	I	TSR-022 (anti-TIM-3) + TSR-042 (anti-PD-1)	OR (Objective Response)
NCT03939975	2	II	Pembrolizumab (anti-PD-1/PD-L1) Nivolumab (anti-PD-1/PD-L1) JS001	AEs Response
NCT02988440	1		PDR001 (anti-PD-1)+ Sorafenib	AEs
NCT02795429	2	II	PDR001 +/− INC280 (c-Met/HGFR inhibitor)	DLT ORR
NCT03474640	1	II	Toripalimab	AEs

**Table 5:** Major trials in HCC involving cell-based immunotherapies.

Name	Phase	Line of Treatment	Strategy	Primary Endpoint
NCT02723942	1/2		CAR-T cell immunotherapy targeting GPC3	Radiological assessment of therapeutic effect
NCT02905188	1	I/II	GPC3-CAR (GLYCAR T cells) + Cytoxan and Fludarabine (lymphodepleting chemotherapy)	DLT (Dose Limiting Toxicity)
NCT03130712	1/2	II	GPC3-CART cells	AEs (Adverse Events)
NCT03198546	1	I	GPC3 and/or TGFβ CAR-T cells	DLT
NCT03013712	1/2		EpCAM-CAR T cells	AEs
NCT03575806	2	II	Autologous Tcm (central memory T cells)	DFS (Disease-free Survival) Clinical Efficacy Safety
NCT02839954	1/2	I	anti-MUC1 CAR-pNK cells (Chimeric Antigen Receptor NK cells with specificity for MUC1)	AEs
NCT04106167			Allogeneic Natural Killer (NK) cells	OS (Overall Survival)
NCT01147380	1		Liver NK cell inoculation	Side Effect
NCT03093688	1/2	II	iNKT (invariant Natural Killer T) cells + PD-1 + CD8^+^ T cells	AEs ORR (Overall Response Rate)
NCT03319459	1	II	FATE-NK100 (donor-derived NK) +/− Cetuximab (EGFR inhibitor)+/− Trastuzumab (anti-HER2)	DLT
NCT02882659	1	II	Dendritic Killer Cell (DKC)	AEs DLT Safety
NCT02886897	1/2	II	Autologous D-CIK (Dendritic and Cytokine-Induced Killer) cells + anti-PD-1 antibody	PFS (Progression Free Survival)
NCT02632006	1/2	I	Pluripotent Killer T Cells expressing antibodies for PD-1	OS
NCT00562666	1		Autologous Gamma-delta T Lymphocytes	AEs
NCT03132792	1	II	Autologous genetically modified AFPᶜ³³² T cells	DLT AEs
NCT03592706	2/3	II	Autologous IKC (Immune Killer Cells)	Change of tumor size PFS
NCT03998033	1/2	II	ET140202 Receptor (+) T cells	AEs RP2D (Recommended Phase 2 Dose)
NCT02678013	3		Cytotoxic T Lymphocytes (CTL)	RFS (Recurrence Free Survival)
NCT03836352	2	II	DPX-Survivac (T cell activating therapy) + Pembrolizumab (anti PD-1/PD-L1) +/− Cyclophosphamide	ORR AEs

**Table 6:** Major trials in HCC involving oncolytic viruses-based immunotherapies.

Name	Phase	Line of Treatment	Strategy	Primary Endpoint
NCT03071094	1/2		Pexastimogene Devacirepvec (Pexa Vec is a vaccinia virus based oncolytic immunotherapy designed to stimulate the immune system following infection and replication within tumor cells) + Nivolumab (anti PD-1/PD-L1)	Safety DLT (Dose Limiting Toxicity) Anti-tumor activity Efficacy
NCT02562755	3		Pexa-Vec + Sorafenib (Raf inhibitor)	OS (Overall Survival)
